# Plasmonic Metasurfaces for Medical Diagnosis Applications: A Review

**DOI:** 10.3390/s22010133

**Published:** 2021-12-25

**Authors:** Zhenbiao Wang, Junjie Chen, Sayed Ali Khan, Fajun Li, Jiaqing Shen, Qilin Duan, Xueying Liu, Jinfeng Zhu

**Affiliations:** 1 Key Laboratory of Electromagnetic Wave Science and Detection Technology, Institute of Electromagnetics and Acoustics, Xiamen University, Xiamen 361005, China; 34320201150180@stu.xmu.edu.cn (Z.W.); sayedali@szu.edu.cn (S.A.K.); 34320191150185@stu.xmu.edu.cn (F.L.); 34320210155961@stu.xmu.edu.cn (J.S.); duanqilin@stu.xmu.edu.cn (Q.D.); 34320190153851@stu.xmu.edu.cn (X.L.); 2 State Key Laboratory of Applied Optics, Changchun Institute of Optics, Fine Mechanics and Physics, Chinese Academy of Sciences, Changchun 130033, China; 3 Analysis and Measurement Center, School of Pharmaceutical Science, Xiamen University, Xiamen 361003, China; chenjunjie@xmu.edu.cn

**Keywords:** plasmonic metasurfaces, biosensing, cancer, COVID-19

## Abstract

Plasmonic metasurfaces have been widely used in biosensing to improve the interaction between light and biomolecules through the effects of near-field confinement. When paired with biofunctionalization, plasmonic metasurface sensing is considered as a viable strategy for improving biomarker detection technologies. In this review, we enumerate the fundamental mechanism of plasmonic metasurfaces sensing and present their detection in human tumors and COVID-19. The advantages of rapid sampling, streamlined processes, high sensitivity, and easy accessibility are highlighted compared with traditional detection techniques. This review is looking forward to assisting scientists in advancing research and developing a new generation of multifunctional biosensors.

## 1. Introduction

Since wood observed the plasmon phenomenon on the subwavelength metal grating in 1902, the development of plasmon has made rapid progress in the last hundred years [1,2,3,4,5,6,7,8,9]. Surface plasmon resonance (SPR) is a collective electronic oscillation phenomenon, which could be classified into two main forms, as surface plasmon polarization (SPP) and localized surface plasmon resonance (LSPR), by their different excitation ways [10]. SPP is commonly produced by shining plane-polarized light onto a continuous metal surface, which can break the diffraction limit and improve the ability to manipulate light on the sub-wavelength scale, making it a promising candidate for the next generation of ultra-miniature integrated photonic circuits and highly sensitive biosensors for information processing [11,12]. The LSPR, on the other hand, is the collective oscillation of electrons at the interface of metal nanoparticles (NPs) irradiated by the excited light of specific frequencies. Due to their sensitivities to the refractive index of the molecular on the media surface, SPR has been widely applied to monitor the molecular binding events on the surface of media [13,14] and gradually became a popular technology in biological detection [15], food safety [16], the medical field [17], and other areas [18]. Compared with the traditional methods of biomedical detections, such as Enzyme-linked immunosorbent assay (ELISA), polymerase chain reaction (PCR), and fluorescence probe-based detection, etc. [19,20,21], SPR has the advantages of being label-free, having a high sensitivity, and real-time dynamic monitoring [22,23,24]. According to different excitation ways, the optical coupling mechanisms of SPR biosensing can be further classified into three categories, namely, prism-based coupling, plasmonic waveguide, and metasurfaces coupling [25,26,27]. The prism coupling detection is greatly dependent on the special apparatus, which consists of an intricate optical and a microfluidic system. Hence, the special detection apparatus used in this method enhances the cost and leads to a large volume, for which the application is hard to be used prevalently. Based on the principle of SPP wave, plasmonic waveguides, especially metal-insulator-metal (MIM) waveguides, which consist of a dielectric core and two metal cladding layers have drawn more attention. Due to the strong localization of SPP modes, they can provide acceptable propagation lengths and ease of manufacture over a very wide wavelength range [28,29,30,31,32].

Plasmonic metasurfaces (nano pillars, bowties, capertures/engravings, nanoholes, etc. [33,34]) are commonly produced by nanofabrication technology, which can be divided into two basic methods bottom-up and top-down methods, according to their different manufacture crafts. Briefly, the former is based on the conditions of a chemical reaction to change the size and shape of the nanomaterials. The latter usually relies on the nanoimprinting and lithography technologies, such as thermal nanoimprinting, ultraviolet nanoimprinting, electron beam lithography (EBL), focused ion beam (FIB) lithography, deep silicon etching, etc. [35]. The plasmonic metasurface is generally fabricated by the novel metals, which have the characteristics of chemical inertness and bio-functional feasibility. Based on the effects of near-field enhancement, it can be used for both labeled and label-free biosensing. For labeled biosensing, plasmonic surface-enhanced detection techniques can be used to improve the optical output of fluorescent measurements [36,37,38]. On the contrary, label-free plasmonic biosensing has attracted more attention, which is feasible on the development of environmentally-friendly portable instruments and demonstrates the potential for point-of-care testing. Compared with the biosensing performance of the important dielectric metasurface counterpart [39,40], plasmonic metasurface has a lower quality (Q) factor of resonance, but their biomolecule sensitivity is usually much higher. This is attributed to the greater near-field concentration and confinement, which is typically within the size scope various biomolecules [10]. The metasurface-based coupling on the specific metal nanostructure is easily detectable by a flexible and tiny device. Besides this, it also has several notable merits compared to prism coupling: wider detection range, better sensing linearity, and more diverse customization. As a result, biomolecular sensors based on metasurfaces are projected to have a greater potential in the clinical diagnosis of a variety of diseases, including malignancies and pandemics.

Cancer is one of the most crucial factors that affecting human life. Noninvasive imaging, endoscopic, and ELISA are usually used in cancer detection [41,42,43,44,45,46,47,48]. However, all of these approaches suffer from several certain limitations involving low sensitivity, inconvenience, and risk of perniciousness, which limit its applicability in the prediagnosis and prognosis of cancer. In the process of cancer detection, tumor markers play an important role. According to the different biological structures, the markers can be divided into protein markers and non-protein markers [49,50,51,52,53,54,55]. To detail the specific application of metasurfaces on the tumor prediagnosis, the protein markers including Carcinoembryonic Antigen sensing (CEA), Prostate-specific antigen (PSA), and other protein tumor indicators, combing with the non-protein markers as exosomes, are illustrated in this review. Compared with traditional detection techniques, the advantages of metasurfaces-based detection in early screening and detection are highlighted.

Additionally, the potential application of metasurfaces sensing in the current pandemic of COVID-19 is also introduced. Up to date, the conventional detection methods involving ELISA and PCR are major techniques to diagnose the infection. However, the complicated operation and long detective period are still major challenges for these methods, especially when the sample number are huge. To improve the screening efficiency of the COVID-19 pandemic and enhance the responsiveness for other pandemics in the future, better and faster diagnostic technologies are urgently needed. The metasurface-based biosensing we have mentioned above is regarded as an interesting alternative approach, where the feasibility in infectious detection has been widely demonstrated in many studies. Thereby, this technology is a promising technique for the rapid diagnosis of viruses.

**Figure 1:**
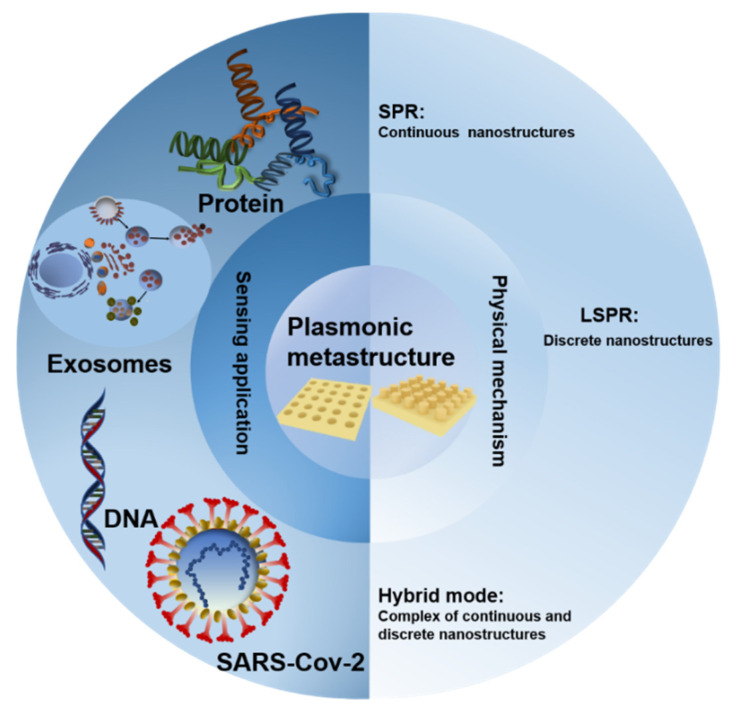
An overview of this review, the center is the typical sensing structure of plasmonic metasurfaces.

To investigate the potential applications of metasurfaces sensing in detail, four sections have been illustrated in this review. In Section 1, we illuminate the physical basics of plasmonic biosensing and demonstrate various typical applications of plasmonic metasurfaces on cancer and COVID-19 detection; the specific applications are summarized in Figure 1. In Section 2, we discuss the principles of metasurfaces which are based on the SPR, LSPR, and hybrid modes. In Section 3, we mainly focus on the application of plasmonic metasurfaces sensors in cancer detection. In Section 4, we introduce the application of continuous periodic metasurfaces and discrete self-assembled metasurfaces in COVID-19 detection. Finally, we discuss how plasmonic surface sensors could be used to detect cancer and viruses.

## 2. Principles of Metasurfaces Biosensing

The light-matter interaction through the evanescent field of SPR is a common mechanism for biomedical analytes detection [56]. The metasurface sensors based on SPR and LSPR are mainly based on the analysis of angle, wavelength, phase, and other plasmon parameters [57,58,59,60,61,62]. Metasurfaces-based SPR refers to a kind of artificial meta-atoms (usually periodic) that are designed to manipulate the amplitude, phase, and polarization of electromagnetic waves [63,64,65,66,67,68]. Compared with the traditional prism coupling-based SPR, the detective facility for metasurfaces-based SPR is easier to be fabricated and the signal is also more stable [69,70,71]. In terms of periodic arrays, the near-field and far-field coupling is utilized to generate resonance with a high quality Q factor on the metasurfaces, and the effect of the collective mode resonance significantly breaks the damping limit of a single metal nanostructure in the dipole approximation. In the case of SPR, it is usually impossible to excite the SPP directly at a perpendicular incidence because the wave vectors do not match directly, whereas, for the periodic array structure (2D grating structure), the diffraction wave will produce a tangential wave vector component provided by the grating of lattice vector, while the waves are incident to the surface of a metal grating. The excitation condition for SPP of periodic grating structure can be written as: [13]

(1) $$\lambda =\frac{a_{0}}{\sqrt{m^{2}+n^{2}}}\sqrt{\frac{\varepsilon_{m}\varepsilon_{d}}{\varepsilon_{m}+\varepsilon_{d}}}.$$

where $\varepsilon_{m}$ and $\varepsilon_{d}$ are metal permittivity and dielectric permittivity, respectively; $a_{0}\;$ is the lattice constant; and  m, n are the scattering orders. On the basis of the equation above (Equation (1)), we can easily draw the positive correlations between the refractive index of the environment changes and resonance wavelength, which is the exact physical mechanism of periodic metasurfaces for biosensing.

In addition to the continuous periodic metasurfaces arrays, some self-assembly (a kind of array with random distribution, which still possesses a certain periodic trend in statistics) metastructures, and other hybrid modes also play an important role in the LSPR domain. LSPR can enhance the electromagnetic field to change its absorption, reflection, and transmission properties for detecting biomolecules [72]. The resonance effect of LSPR can be regarded as a metal spherical nanoparticle with a radius of *a* (*a* ≪ λ) under the action of an electromagnetic field. By solving the Laplace equation with a boundary condition, combing with metal Drude mode formula, then ignoring the damping effect, the resonance frequency $w_{l}$ of LSPR can be defined as [73]:

(2) $$w_{l}=w_{P}{\left[\frac{l}{\varepsilon_{d}\left(l+1\right)+l}\right]}^{\frac{1}{2}}$$

where $\varepsilon_{d}\;$and $w_{p}$ are the permittivity of environment and plasma frequency, respectively, and l  is the angular momentum of the resonant mode. This is similar to the sensing mechanism of the SPR. The variation of background permittivity $\varepsilon_{d}$ will lead to the spectral shift Δw (correspondingly $\Delta_{\lambda }$ for wavelength) of LSPR, which is the basic sensing mechanism.

For either SPR or LSPR, bulk refractive index sensitivity (*S*), the figure of merit (*FOM*), and limit of detection (*LOD*) are very important elements to metasurface-based sensor performance. *S* is an important parameter determined by the environmental refractive index changes, derived from the molecular interaction on the surface of metastructures. Thus, it is used to evaluate the sensing capability of an optical sensor. The correspondence between *S* and *n* can be described according to Equation (3): [68]

(3) $$s=\frac{dA}{dn}$$

where *A* denotes the measured physical parameters (wavelength, angle, or spectral intensity), and *n* is the refractive index. Deuterium lamps, halogen lamps, or their combined light sources, paired with optical fibers and spectrometers, are commonly utilized for plasmonic sensors. Changes in the wavelength, angle, and intensity of the spectrum are usually caused by changes in the refractive index n of the analyte. The sensitivity of plasmon sensors can range from 50 nm/RIU (refractive index unit) to 30,000 nm/RIU (refractive index unit) depending on structural design and material selection [74].

*FOM* is another important parameter that is utilized to define the sensor’s ability to respond to changes of small refractive index. For plasmonic plane metal structures, *FOM* is usually not high due to intrinsic losses, but for the metastructures, the intrinsic losses can be reduced by using different materials and structures. The relationship between *FOM* and *S* can be defined as [75]:

(4) $$FOM=\frac{S}{FWHM}$$

where *FWHM* represents the full width at half maximum of resonance spectra.

*LOD* is the third sensing parameter, which is mainly determined by sensitivity and noise level. The relationship between *LOD* and *S* can be written as [73,75]:

(5) $$LOD=m\frac{\sigma_{blank}}{S}$$

where *m* is a numerical factor, and $\sigma_{blank}$ is the standard deviation of the blank measures. The *LOD* is mainly determined by sensitivity and noise level, so it can be improved with low noise detectors and light sources.

## 3. Tumor Marker Screening Based on Plasmonic Metasurfaces

**Figure 2:**
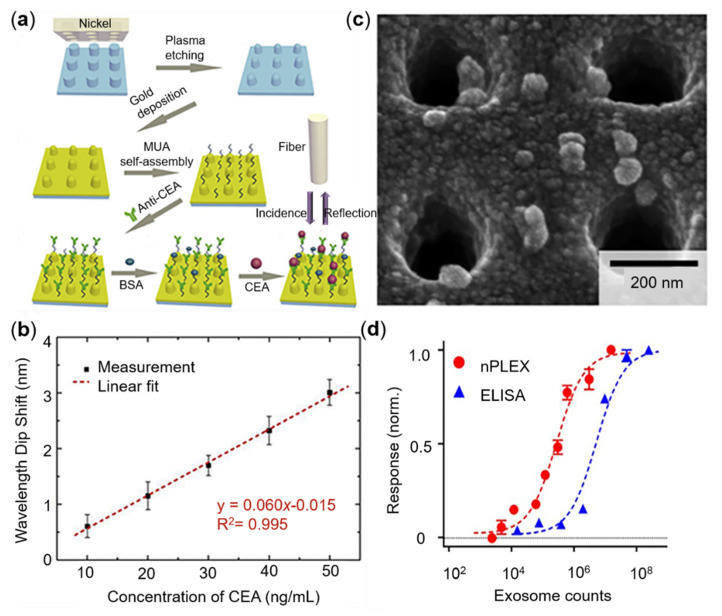
Functionalization of different nanostructures and sensing performance. (**a**) Biofunctionalization of periodic nanorods. (**b**) The linear fitting relationship between wavelength dip shift migration and CEA concentration. (**c**) Scanning electron microscope (SEM) image of exosomes captured by functionalized nanohole arrays. (**d**) Comparison of exosome detection sensitivity between nanohole chip and ELISA. Reprinted (**a**,**b**) with permission from Reference [76]. Reprinted (**c**,**d**) with permission from Reference [77].

The biosensor based on the SPR is an emerging platform for disease biomarkers detection. Comparing with the ELISA etc., the approach can provide a non-invasive, real-time, label-free, and rapid detection of cancer markers. To gain a precise screening, the selection of suitable cancer biomarkers is very important. According to the general classification of the biological properties of biomarkers, this section focuses on the detailed screening and detection of protein biomarkers, which are widely used in clinical practice, involving CEA sensing, PSA, and exosomes (Figure 2).

### 3.1. CEA Sensing

CEA is a cell membrane glycoprotein expressed by normal mucocytes. The normal content of CEA in adults’ bloodstream is approximate 2.5 ng/mL, while the content in tumor patients usually surges to 100 ng/mL or more. The elevation of CEA in serum commonly indicates the possibility of colorectal cancer, gastric cancer, breast cancer, lung cancer, and ovarian cancer [78]. Therefore, as a conventional broad-spectrum tumor marker, CEA detection has been widely studied in the SPR field. Zhu et al. have produced a cyclical hard gold nanohole array by using nanoimprint and oxygen plasma etching technology [79]. Combining with the soft nano-imprinting lithography, microfluidics, antibody functionalization, and mobile optical spectroscopy, they established a cost-effective plasmonic metasurfaces immune sensing platform, which realized the portable detection of CEA. By plotting the changes of wavelength dip on the event of CEA binding, the shift of wavelength dip against different CEA concentrations will decrease or increase linearly in a special range. The real CEA content in the serum of cancer patients was utilized to assess the detection, and the result demonstrated a high accuracy with a very small error, referring to the real content. The sensitivity is up to 490.2 nm/RIU, and the limit of detection (*LOD*) was 5 ng/mL, four times lower than the threshold of 20 ng/mL of CEA detection. Based on the gold nanohole array in the visible light regime, they achieved stability and high-sensitivity detection by using the microfluidic technology, then provided a new solution for portable medical and real-time diagnosis [79]. The manufacturing process of the hard substrate is complex, which is inconvenient to the application of the smart devices. To simplify the process, Zhu et al. further designed a flexible periodical nanopillar. By coating the gold film with the polycarbonate substrate IPS (Polycarbonate), the sensitivity and *LOD* of the bio-functional IPS were boosted to 454.4 nm/RIU and 5 ng/mL, respectively; illustrations are shown in Figure 2a,b. In addition, the manufacturing steps and cost are also reduced. This technology is suitable for large-scale production and commercialization and shows the potential for clinical and future applications in flexible wearable devices [76].

### 3.2. PSA Sensing

**Figure 3:**
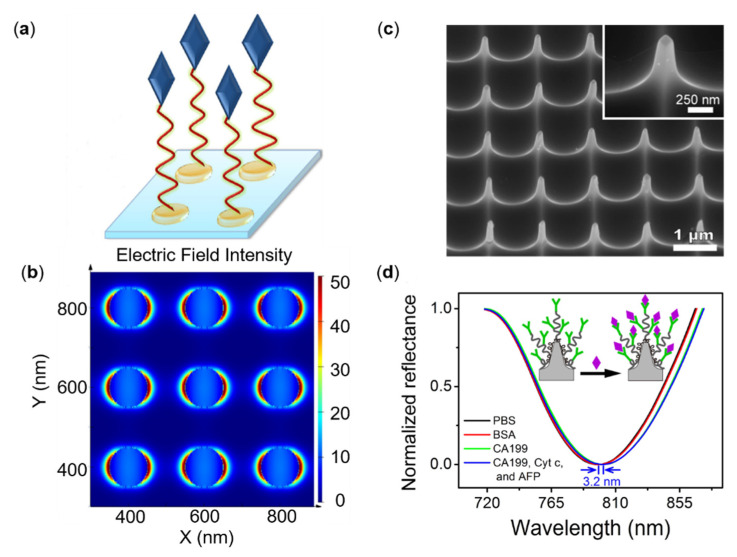
Different nanostructures and PSA sensing performance. (**a**) Biorecognition process of PSA based on gold nano disks. (**b**) The distribution of the electric field intensity at resonance around gold nanodisk array by FDTD simulation. (**c**) Scanning electron microscope (SEM) image (side view) of aluminum nanopyramid array. (**d**) Reflectance spectra for detecting CA199 based on the anti-CA199 modified Al nanopyramid array by specific interaction in different solutions; Reprinted (**a**,**b**) with permission from Reference [83]. Reprinted (**c**,**d**) with permission from Reference [87].

PSA is a glycoprotein mainly secreted by the prostate cells. However, it is also a tumor marker, usually utilized for carcinoma diagnosis. The diagnostic gray of PSA is 4.0–10 ng/mL, and when the concentration is higher than 10 ng/mL in serum, the risk of prostate cancer is increased [80]. Therefore, the accurate quantization of PSA levels is very important for the early diagnosis of cancer [81,82]. Khan et al. provided an idea for monitoring other biological interactions by virtue of DNA aptamers. Due to the advantages of strong versatility, resistance to degeneration, and substrate recovery [83], the DNA aptamer-functionalized gold nanodisk array achieved a bulk sensitivity of 113 nm/RIU based on LSPR extinction spectroscopy, and the *LOD* is as low as 1.49 ng/mL with the dynamic range from 1.7 to 20.4 ng/mL in PSA detection [83] as shown in Figure 3a,b. For realizing miniaturization and integration, Lin et al. utilized a kind of reusable gold nanodisk arrays as a nanoprobe at the fiber end used by the Electron Beam Lithography (EBL) and metal peel technology, achieving 100 FG/mL (3 fM) *LOD* for the free F-PSA [84]; Couture et al. designed a periodic hexagonal nanohole array through lithography technology on a 4-inch glass wafer. In comparison to single channel measurements with nanohole arrays fabricated with nanosphere lithography, the nanohole array sensors greatly enhanced the signal-to-noise ratio of the plasmonic signal and precision of the measurements with the multiwell plate system for achieving a low antibody detection range. Due to the small molecular weight (28 kDa) [85], PSA usually requires a secondary antibody for the amplification of the plasmonic signal. By using sandwich determination, the *LOD* of the proposed sensor was lowered to 0.1 nM [86].

### 3.3. Other Protein Tumor Markers Sensing

Despite of the CEA and PSA, other tumor markers including Tumor necrosis factor-alpha (TNF-α), carbohydrate antigen (CA199), and alpha-fetoprotein (AFP) were studied by the biosensing performed on either different material or shaped metasurfaces. For example, Jin et al. designed a gold nanometer mushroom array. Based on the interaction between the Wood anomaly and local surface plasmon, the refractive index sensor with quality factor is up to$\;10^{8}$, which approaches the upper limit of the theoretical prediction of a standard PSPR sensor. The array achieves the bulk sensitivity of 1015 nm/RIU and was also validated by alpha-fetoprotein detection. Furthermore, the observed *LOD* reaches 15 ng/mL [88].

TNF-α is an inflammation-related protein, which plays an important role in disease diagnosis and prediction. Monteiro et al. used a gold nanohole array by transmitted light intensity monitoring to achieve a sensitivity of 4000–5300 IU/RIU and a *LOD* of 17 pg/mL [89]. Aluminum has an advantage over the classic noble metal induction process because of its natural abundances, low cost, ease of processing and large-scale manufacturing, and ease of processing with a range of technologies, including complementary metal oxide semiconductor process. However, aluminum’s plasmonic mode is limited by its UV-green wavelength, poor refractive index, facile surface oxidation, and structure dependence, which hinder the application of aluminum in plasmonic biosensing. Zhou et al. achieved a rapid detection of tumor marker CA199 via a uniform quasi-three-dimensional aluminum nanocone array, which could generate the tunable UV-visible-near-infrared plasmons by changing the incident angle and achieving a *LOD* of 29 ng/mL in the air [87]. More interestingly, In the immunodetection of the bovine serum protein (BSA) protein antibody, Zhu et al. synthesized nanoimprint printing, oxygen plasma etching, metal film deposition, oxygen plasma passivation, and other processes to prepare 2-inch aluminum nanocrystals on a flexible polymer substrate with a detection limit of 1 pg/mL. The sensitivity of aluminum-based metasurfaces is two orders of magnitude higher than that of gold metasurfaces with the same structure, demonstrating improved biomolecular immunodetection ability and clinical application potential [90]. Differing from the conventional detection in the visible band, the THz metamaterial has a precise and tunable vibration frequency, which is consistent with the vibration frequency of some important tumor marker molecules, but its water absorption phenomenon is a major obstacle that hinders the micro-detection of tumor marker antigen antibodies. Geng et al. and co-workers suggested overcoming the issue of water absorption and gained a 14.2 GHz resonance displacement (0.02524 ug/mL) in tumor marker AFP detection by using a metal nano-cracked ring resonator and Polydimethylsiloxane (PDMS) microchannel [91]. Furthermore, by refining the metamaterial’s structure and lowering the matrix’s dielectric constant, the sensitivity of specific cancer biomarkers can be boosted even more. This method has strong application potential for the special recognition of early cancer molecules.

### 3.4. Tumor-Derived Exosome Sensing

Exosomes are a kind of extracellular microvesicle secreted by miscellaneous cells in whole human organism and widely exist in blood, urine, saliva, and even breast milk [92]. Due to its ability to transport the molecular contents from the originated cells to the targeted cell, exosome is also notorious for the role of an accomplice in tumor progress. The abnormal increase of exosomes is always found in those patients who suffer from a malignant tumor. Thereby, exosomes are regarded as a potential biomarker in cancer diagnosis. In the absence of biopsy, the capture of exosomes containing molecular information from their parent tumor cells can be used as a novel means to predict and diagnose cancer [93]. However, traditional methods of exosome detection based on ultracentrifugation, western blotting, and ELISA require complicated sample handling and professional experiment operation [94], which limit the detection to realize miniaturization and portability. Recently, the modulated metasurfaces-based detection provides a new idea for fast and portable detection of exosomes. Lee et al. proposed a nanoplasmonic exosomal sensing (nPLEX) technique based on periodic nanohole arrays; the illustration is shown in Figure 2c,d. The nanohole structures greatly improve the detection sensitivity on exosomes by limiting the surface electromagnetic field and enhancing the evanescent field referring to the exosome size ranges [77,95,96]. By employing a complementary metal-oxide-semiconductor (CMOS) apparatus, the binding of exosomes to the nanohole surface could be easily reflected by the phase difference or intensity variation, which is induced by the shift of the plasmon resonance signal. The nPLEX array with 36 sensing units can synchronously support 12 potential exosome markers detection in parallel, achieving the LOD of approximately 3000 exosomes (670 aM) in 30 min. Furthermore, the nPLEX platform can be enhanced further by the secondary labeling of Au nanoparticles. The nPLEX chip can be scaled up to improve throughput in clinical applications for high-throughput clinical diagnosis of pancreatic malignancy by multi-marker extracellular vesicle, EV [95,96].

**Figure 4:**
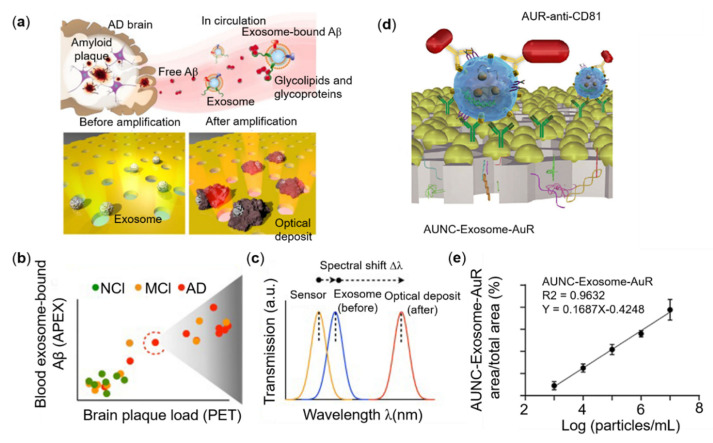
Performance of different nanostructures and sensing performance for exosomes. (**a**) Exosomes associate with Aβ proteins. The Aβ protein, the main component of amyloid plaques found in AD brain pathology, is released into the extracellular space. Exosomes are nano-scale outer cell membrane vesicles secreted by mammalian cells. Exosomes bind to the released Aβ protein through their surface glycoproteins and glycolipids (**b**) The APEX platform was used to measure exosomal-bound Aβ in blood samples of Alzheimer’s disease (AD), mild cognitive impairment (MCI), and no cognitive impairment (NCI) control groups. The blood measurement results are correlated with the corresponding PET imaging of cerebral amyloid plaque deposition. (**c**) A representative schematic diagram of the change transmission spectrum with APEX magnification. The APEX platform monitors the specific exosome binding (before) and the subsequent amplification spectrum (after) transmission spectrum shift (Δλ). a.u arbitrary unit. (**d**) Schematic illustration of in-situ detection of exosome (**e**) Correlation of AuNC-Exosome-AuR signal ratio against to exosome concentration. Reprinted (**a**,**c**) with permission from Reference [97]. Reprinted (**d**,**e**) with permission from Reference [98].

Based on nanohole arrays, Shao et al. measured different populations of circulating amyloid β (Aβ) proteins—exosome-bound vs. unbound—directly from blood. The local optical deposits and double-layer plasma nanostructures for in-situ enzyme conversion were used to achieve high sensitivity. More interestingly, the multi-channel population analysis achieves high sensitivity (about 200 exosomes) and can attain the co-localization of multiple targets in exosomes. This shows the important role of exosomes in the diagnosis of Alzheimer’s [97]. Illustrations are shown in Figure 4a–c. Liu et al. designed an integrated microfluidic device of nanoporous gold (Au) with modified membrane nanoclusters to capture antibodies. The second antibody-coupled gold nanorod probe was loaded under a dark field microscope to identify and quantify the specificity of lung cancer. Exosomes can be used to isolate and detect on-site lung cancer-specific exosomes collected from the patient’s urine. Due to resonance Rayleigh scattering, the complex produces a significant scattering wavelength shift and increases the scattering intensity, which enables the ultra-sensitive detection of exosomes with a *LOD* less than 1000 particles/mL [98], as shown in Figure 4d,e. 

In addition to the conventional two-dimensional nanohole arrays, Pang et al. studied and compared two-dimensional nanoholes, quasi-three-dimensional nanohole arrays, and 3D photonic crystal structures. Due to the hybrid coupling of LSPR and Fabry–Perot cavity modes, the quasi-three-dimensional hole structure was stronger. The electromagnetic field is relative to the two-dimensional nanopore. In the comparison of these three structures, it is found that the 3D photonic crystal structure based on plasmonics and photonic crystal modes has a higher sensitivity, reaching 1376 nm RIU−1. Peak shift increased to 102 nm as exosome concentration increased to 1 × 10^11^ particles per mL. It shows the potential of 3D photonic crystal structure in the process of exosome detection [99]. Performances of various nanostructured arrays for cancer detection are summarized in Table 1.

## 4. COVID-19 Sensing Based on Plasmonic Metastructures

Since the beginning of the COVID-19 pandemic, infectious disease has brought a huge health threat to the whole world [102]. The rapid screening and insulation of the infectious person from the healthy population is still a major means of pandemic control. Many reviews have introduced the development of COVID-19 and its detection methods [103,104,105,106,107,108,109,110,111,112,113,114,115,116,117,118,119]. Nucleic acid amplification and serological testing are two common methods of COVID-19 detection [120,121,122,123,124,125]. Nucleic acid detection based on two steps of operations consists of reverse transcription-polymerase chain reaction (RT-PCR) and real-time fluorescence quantify PCR (Q-PCR) is still a gold standard for the infection screening [126,127,128,129]. However, this method has the disadvantages of long period detection time, is expensive, and requires professional operation [115]. Serological test including ELISA and later flow assay (LFA) is another common detection method of pathogen infection, which can rapidly identify an infectious individual who has the immune response [130,131,132,133,134,135,136]. Similar to nucleic acid detection, either ELISA or LFA also suffer numerous complicated operations, including antibody coating, blotting, longtime incubation, scrubbing, and chemical reaction, which limit its application to POC [134]. Due to the unavoidable disadvantages that exist in nucleic acid detection and serological tests, a rapid detection method with real-time, unlabeled, and miniaturization is urgently needed [137,138,139]. The metasurfaces-based SPR detection is expected to be an alternative but more promising approach, which could achieve a real-time, unlabeled, and rapid detection on COVID-19.

### 4.1. Detection Based on Metasurfaces

**Figure 5:**
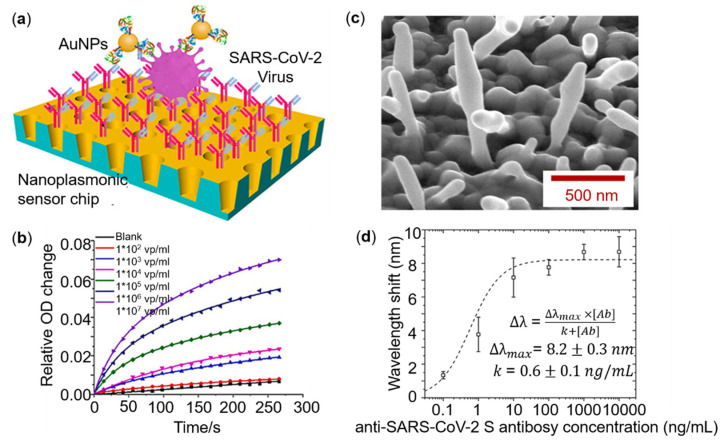
Different structures and sensing properties of SARS-CoV-2 detection. (**a**) Schematic diagram of nanocup array for COVID-19 detection. (**b**) SARS-CoV-2 mAbs labeled AuNP enhanced binding curves with different concentrations of the SARS-CoV-2 pseudo virus over the range 0–1.0 × 10^7^ vp/mL. (**c**) SEM diagram of nano spikes structure. (**d**) LSPR responses at different anti-SARS-CoV-2 spike protein antibody concentrations. Reprinted (**a**,**b**) with permission from Reference [140]. Reprinted (**c**,**d**) with permission from Reference [141].

Recently, some researchers have developed diversified detection of SARS-CoV-2 based on SPR technology [140,141], as shown in Figure 5a–d. Liu et al. used gold discrete nanocup arrays to detect the novel coronavirus spike protein and gained a *LOD* of 370 vp/mL with a decent detective range from 0 to 10^7^ based on a label-free LSPR biosensor with an extraordinary optical transmission (EOT) effect. Results are illustrated in Figure 5a,b. In addition, gold nanoparticles are used to enhance the sensitivity of the sensor, quantify the low concentration analysis in a solution with limited diffusion conditions, and amplify the optical signal to shorten the detection time. Similar fast and sensitive detection capabilities were demonstrated using low-cost handheld optical devices controlled by a smartphone application (APP) program. The *LOD* of SARS-CoV-2 pseudo viruses in hand-held systems was about 4000 virus particles within 15 min [140]. Yanik et al. developed a kind of plasmonic nanohole arrays, in which the antibody of the spike protein of SARS-CoV-2 was initially immobilized on the surface. By capturing the spike protein, the whole viruses were suspended into the nanohole arrays of grating, and directly resulted in the oscillator redshift of the resonant frequency, when the grating coupling of the incident light on the surface. On the basis of this, small (vesicular stomatitis virus and pseudo Ebola-virus) and large (vaccinia virus) enveloped viruses can be non-destructively detected [142].

### 4.2. Detection Based on Self-Assembled Metastructures

Self-assembled metastructures are another nascent metasurfaces, which are also able to be used for virus detection. Funari et al. manufactured gold nanospikes metasurfaces based on electrodeposition technology combined with optical probes to form an optical microfluidic sensing platform. Based on the changes in the local environmental refractive index caused by the interaction between the SARS-COV-2 spike protein and the antibody in the diluted human serum, the shift of the LSPR resonance peak was detected, and a detection concentration of 0.08 ng/mL was achieved. The diagnostic platform demonstrates the potential to complement existing serological assays and improve COVID-19 diagnosis; the results are shown in Figure 5c,d [141]. Furthermore, a significantly higher sensitivity (picomolar) was achieved by Qiu et al. By combining the hybrid effect of plasma photothermal and LSPR, they enhanced the LOD of virus sequences to 0.22 pM, providing a new idea for the detection of COVID-19 [143,144]. On the other hand, the self-assembling nanoparticles made by noble metal are also used to amplify optical signals in the biosensing process. Das et al. gained a LOD of the spike of SARS-COV-2 to 111.11 deg/RIU by using a gold nanorod nanoparticle with a Kretschmann prism configuration [145]; Parikshit et al. proposed the LSPR colorimetric sensor composed of gold nanoparticles. The assay is based on the specific targeting and binding ability of antisense oligonucleotides to the N gene of the SARS-CoV-2 virus genome. By immobilizing the antisense oligonucleotides to gold nanoparticles, the SPR signal derived from binding events was significantly enhanced. In the presence of SARS-CoV-2 target RNA sequence, the AuNP at the closure of ASOs (antisense oligonucleotides) modified by mercaptan was selectively aggregated, which induces a redshift of about 40 nm in its absorption of SPP spectrum. Based on this method, positive cases infected by SARS-CoV-2 were diagnosed within 10 min [146]. Ahmadivand et al. designed a ring plasma cell sensor to detect the S protein. The assay illustrates an excellent sensitivity with an extreme LOD of 4.2 fM by antibody-AuNP complex. It is also worth mentioning that the transmission spectrum of the meta-sensor enables the movement of a polarized beam that excites the etheric Hertz frequency, which confers a promising application in the point of care (POC) filed [147]. Cheong et al. developed a compact nano PCR system that enables SARS-CoV-2 RNA to be detected by a portable device. Rapid thermal cycling (via plasma heating of magneto-plasma nanoparticles) and in situ fluorescence detection was enabled after the magnetic removal of nanoparticles. By using this approach, three samples can be measured simultaneously within 17 min, and the LOD was as low as 3.2 gene/uL [148]. Gao et al. combined three modes of colorimetry, SERS, and fluorescence detection to detect the RNA of the COVID-19 virus, achieving a LOD of 160 fM after 40 min of incubation [149]. The typical sensing performance of metasurfaces devices for COVID-19 is summarized in Table 2.

## 5. Conclusions and Future Trend

In this paper, we briefly review the applications of plasmonic metasurfaces in biosensing. We discuss the physical sensing mechanisms of the metasurface, focusing on recent applications of cancer detection and COVID-19 detection.

As for the future development of plasmonic metasurface sensing, we believe that there is still a great part room for improvement of either detection devices or plasmon materialization and commercialization. At present, plasmonic-based disease detection is limited to serum, which needed a complicated preprocessing in the professional laboratory before detection. However, in order to achieve the POCT, by capturing and manipulating light on the chip, and manipulating discrete chemical samples on micron and sub-micron scale chip structures to enhance the function of the chip chemical platform, it may further promote the development of plasmon miniaturization [150,151,152,153,154,155,156] and more accessible sample involving whole blood, or even involving sweat, saliva, and urine may be a better choice. This will also bring a greater challenge to portable medical applications in health diagnoses. With the rapid development of optical technology, in terms of devices with complicated functions, small-scale and high-integration density is expected to be more flexible and wearable [157], and plasmonic biosensing can be used in wearable devices to detect those complicated samples in real-time.

The Biacore series of commercialized plasmonic biosensors (such as the American General Electric Company’s Biacore 8K series) are based on flat metal film and uses the SPP effect activated by evanescent waves. It is mainly used to analyze the binding kinetics and affinity tests of biomolecules. The commercialization of plasmon metasurfaces instant detection equipment still needs a certain process. There are no mature instruments in the market. In the future, it may be necessary to solve the problem of large-scale manufacturing of plasmon chips while maintaining cost control and promoting the commercialization process well.

On the other hand, metamaterial is another approach to improving detection efficiency and lowering the commercial cost, so the development of novel materials and structural design will boost detection technology [158,159,160,161,162]. Conventional noble metals are commonly used in plasmonic sensing; however, an inherent feature of optical loss is that it is hard to be overcome. TiN is a novel material which has been recently used in plasmonic study due to its stable chemical property and free carrier concentration close to that of gold. The nonlinear optical response based on free carriers provides a wide spectral response range. More intriguingly, TiN has one order of magnitude better stability than gold and other noble metal plasmon materials. Furthermore, TiN is a low cost and easily available material, which confers its promising commercial application in plasmonic biosensing. Apart from the material selection, the structural design of the medium is also an alternative optimization scheme for plasmonic biosensing [163,164]. The required metastructure is reversely developed in conjunction with artificial intelligence to achieve molecular customized sensing during biosensing and make efficient use of the electromagnetic field’s improved hot spot [165,166,167,168].

## Figures and Tables

**Table 1:** Performances of tumor markers based on metasurfaces.

Metastructure	Analytes	Bulk Sensitivity	LOD	Reference
Nanohole	CEA	490.2 nm/RIU	5 ng/mL	[79]
Nanopillar	CEA	454.4 nm/RIU	5 ng/mL	[76]
Nanocup	CEA	800 ΔT%/RIU	10 ng/mL	[100]
Nanodisk	PSA	113 nm/RIU	1.49 ng/mL	[83]
Nonohole	PSA	/	0.1 nM	[86]
Nanohole	CD24	/	0.18 ng/μL	[77]
Nanopyramid	CA199	819 nm/RIU	29 ng/mL	[87]
Nanomushroom	AFP	1015 nm/RIU	15 ng/mL	[88]
Nanosplit-ring	AFP	/	0.02524 μg/mL	[91]
Nanohole	TNF-α	4000–5300 IU/RIU	17 pg/mL	[89]
Nanohole	Aβ	/	200 exosomes	[97]
Nanoporosity	CD-63	/	1 particle/μL	[98]
Nanohole	Exosomes	1736 nm/RIU	/	[99]
Nanopillar	CEA	/	5 ng/mL	[40]
Nanodisk	PSA	/	1.6 ng/mL	[101]

**Table 2:** Performance of COVID-19 based on metasurfaces.

Metastructure	Analytes	LOD	Reference
Nanospike	S protein	0.08 ng/mL	[141]
Nanocup	S protein	370 vp/mL	[140]
Nanoisland	SARS-CoV-2	0.22 pM	[143,144]
Nanorod	S protein	111.11 deg/RIU	[145]
Nanoparticle	N gene	0.18 ng/uL	[146]
Toroidal metasurface/nanoparticle	S protein	4.2 fM	[147]
Nanohole	S protein	/	[142]
Nanoparticle	RNA	160 fM	[149]
Nanoparticle	RNA	3.2 gene/uL	[148]

## Data Availability

Not applicable.
